# Simultaneous Interrogation of Cancer Omics to Identify Subtypes With Significant Clinical Differences

**DOI:** 10.3389/fgene.2019.00236

**Published:** 2019-03-28

**Authors:** Aodan Xu, Jiazhou Chen, Hong Peng, GuoQiang Han, Hongmin Cai

**Affiliations:** School of Computer Science and Engineering, South China University of Technology, Guangzhou, China

**Keywords:** similarity integration, omics data, survival analysis, DNA methylation, gene expression, miRNA

## Abstract

Recent advances in high-throughput sequencing have accelerated the accumulation of omics data on the same tumor tissue from multiple sources. Intensive study of multi-omics integration on tumor samples can stimulate progress in precision medicine and is promising in detecting potential biomarkers. However, current methods are restricted owing to highly unbalanced dimensions of omics data or difficulty in assigning weights between different data sources. Therefore, the appropriate approximation and constraints of integrated targets remain a major challenge. In this paper, we proposed an omics data integration method, named high-order path elucidated similarity (HOPES). HOPES fuses the similarities derived from various omics data sources to solve the dimensional discrepancy, and progressively elucidate the similarities from each type of omics data into an integrated similarity with various high-order connected paths. Through a series of incremental constraints for commonality, HOPES can take both specificity of single data and consistency between different data types into consideration. The fused similarity matrix gives global insight into patients' correlation and efficiently distinguishes subgroups. We tested the performance of HOPES on both a simulated dataset and several empirical tumor datasets. The test datasets contain three omics types including gene expression, DNA methylation, and microRNA data for five different TCGA cancer projects. Our method was shown to achieve superior accuracy and high robustness compared with several benchmark methods on simulated data. Further experiments on five cancer datasets demonstrated that HOPES achieved superior performances in cancer classification. The stratified subgroups were shown to have statistically significant differences in survival. We further located and identified the key genes, methylation sites, and microRNAs within each subgroup. They were shown to achieve high potential prognostic value and were enriched in many cancer-related biological processes or pathways.

## 1. Introduction

In current clinical practice, cancer is typically categorized based on its tissue source and pathological histology. However, cancer is also known as a well-characterized pathological system among the molecular level. Most cancers emerge along with complex molecular alterations at the germ and/or somatic level (Kristensen et al., 2014). Molecule-level cancer re-classification and subtyping based on genome-scale data sets can act as a sally port for precision oncology (Wu et al., 2017), such as for evaluating the metastatic potential of patients and selecting the most promising treatment (Forbes et al., 2010). Although enormous quantities of molecular data have been accumulated from various cancer profiling projects, for example, the Catalog of Somatic Mutations in Cancer (COSMIC) database (Forbes et al., 2008), the International Cancer Genome Consortium (ICGC) (International Cancer Genome Consortium et al., 2010), and The Cancer Genome Atlas (TCGA) (Weinstein et al., 2013), interpreting such data is difficult. In recent years, many sophisticated statistical and mathematical models have been proposed to analyze biological data, most of which are based on a single data type (e.g., gene expression, methylation). However, all biological mechanisms consist of multiple molecular phenomena and genomes exhibit variation owing to gene mutations, epigenetic changes, individual differences and environmental influences. It is difficult for conventional analysis based on data from a single genome to capture the heterogeneity of all biological processes and clearly differentiate phenotypes. Thus, the focus has now been shifted to how to integrate multi-omics to achieve more promising and stable cancer classification results.

To perform such simultaneous interrogation, there are two major challenges. First, distinct omics data are heterogeneous in scale, dimension, and quality, and such heterogeneity requires subtle processing. Second, there are internal relationships between single data layers (e.g., the promoter DNA methylation may suppress expression). As such, information on these regulatory patterns can improve our integrated analysis. Existing methods can be roughly divided into three categories based on their methodology: latent variable representation methods, probabilistic modeling methods, and network-based methods (Huang et al., 2017; Rappoport and Shamir, 2018). Latent variable representation are mainly committed to mapping diverse features from different data types into a shared low-dimension common space under the assumption that a set of latent variables is shared across multi-omics data. For example, iCluster+ employs an expectation-maximization (EM) algorithm to build regularized regression in modeling latent variables and observed data (Mo et al., 2013). A joint non-negative matrix factorization (jNMF) method is used to detect the shared characteristic space (Zhang et al., 2012). A moCluster algorithm can define a joint latent variable using the modified consensus PCA (CPCA) (Meng et al., 2015). The major drawback of these methods is that, when dimensions and variances of different omics datasets differ greatly, the basic assumption may be unexplainable. The unobserved latent variables possess little biological meaning and have far fewer dimensions than original spaces. Probabilistic models always presume different prior distributions of multi-omics data, constructing a mixture model, and then estimate the parameters and mixture ratios. For instance, a Beta-Gaussian mixture model can integrate gene expression data and protein-DNA binding probabilities into a single probabilistic modeling framework (Dai et al., 2009). Except for modeling original data, we can also model the probability of clusters distribution on the local and global level using the hierarchical Dirichlet mixture model (Gabasova et al., 2017). However, the accuracy relies heavily on the inherent distribution of data and overfitting may occur when sample size far less than features. Instead of searching common latent variables in measurement space, network-based methods begin with each single data layer and propagate information through interactions between samples to construct a global graph structure. A previous work named similarity network fusion (SNF) (Wang et al., 2014) follows this route using the message-passing theory to fuse similarities of each available data type into one network by iteratively updates every network as the similarity matrix product of a single layer and the average of the rest layers. Network structure can effectively handle differences in dimension and scale. However, the main difficulty lies in how to determine the contributions of each local pattern and how to interpret the clustering result in terms of the original features. Hence, there are still-strong demands for efficient and precise multi-omics data integration methods that can overcome the dimension variance and heterogeneous scale.

In this paper, we proposed a method to interrogate omics data simultaneously to achieve multi-scale cancer subtyping. The proposed high-order path elucidated similarity (HOPES) integrates the similarities for each type of omics data into a unified and stable one, thus achieving a simplified link of the underlying mechanism of various types of expression. We modeled integrated similarity as the approximation to various high-order paths across each local dataset, the progressively increased high-order path can represent different consistency requirements. We especially emphasized interaction within each pair of local layers rather than updates using a single layer and average of the rest layers. HOPES models such similarity integration as a minimization problem consist of three subobjective functions, for which an efficient numerical algorithm was designed to obtain the solution. Through the optimization procedure, we strengthened the strong correlation between patients and removed the weak ties mainly caused by noise. Thereby, we successfully subtype cancers with significant clinical differences. Real experiments on five cancer projects of TCGA and a normal control set for cancer diagnosis and prognosis tasks demonstrated the excellent performance of HOPES in subtyping and identifying key oncogenesis pathway. The subsequent biological analysis of the resulted key pathway was shown to possess potential prognostic value and biological significance.

## 2. Materials and Methods

### 2.1. Tumor Datasets With Comprehensive Omics Measurements

We tested the proposed HOPES on five distinct tumor datasets, downloaded from TCGA. The tested samples consisted of five tumor types: glioblastoma multiforme (GBM), lung squamous cell carcinoma (LUSC), kidney renal clear cell carcinoma (KIRC), colon adenocarcinoma (COAD), and a cervical cancer dataset (CESC). Each tumor was measured by DNA methylation, gene expression, and miRNA expression. The overall survival information corresponding to each sample was also considered. The first four projects were the same as the experimental data obtained in a previous study (Wang et al., 2014). The gene expression data for GBM and LUSC were collected using the Broad Institute HT-HG-U133A platform, while COAD was collected by the UNC-Agilent-G4502A-07 platform, and KIRC by the UNC-Illumina-Hiseq-RNASeq platform. The miRNA expression data for GBM were collected by the UNC-miRNA-8X15K platform, while those for LUSC, KIRC, and COAD were collected by the BCGSC-Illumina-GA-miRNAseq. The methylation for GBM was analyzed by the JHU-USC-Illumina-DNA-Methylation platform, while for the others the JHU-USC-Human-Methylation-27 platform was used. The fifth CESC dataset contains data on clinical and pathological features, genomic alterations, DNA methylation profiles, and RNA and proteomic signatures, and is available from TCGA (Cancer Genome Atlas Research Network et al., 2017). We collected gene expression profiles, DNA methylation expression, miRNA expression, and clinical data from the Broad Institute TCGA Genome Data Analysis Center (Broad Institute TCGA Genome Data Analysis Center, 2016). A total of 284 samples with these four types of data were included in the study. For each data type, we removed signatures with a missing rate among all of the samples higher than 20%. For the remaining missing-value data, a K-nearest neighbor (KNN) imputation (Troyanskaya et al., 2001) scheme was used to complement it by filling the empty area with the mean value of non-empty neighbors. Finally, we normalized each dataset across samples and obtained a gene expression dataset of 20,118 genes, a methylation dataset of 396,065 CpG sites, and a miRNA dataset of 885 miRNAs. To reduce computational cost, for analysis involving methylation data, the 1,000 most variable CPG sites based on the standard deviation of beta values were selected.

### 2.2. Comparative Healthy Dataset as a Control

Besides the tumor samples, we also prepared normal samples as a control set to evaluate the capacity for using HOPES in diagnosis. A few healthy cases with data on gene expression, methylation, and miRNA expression are also included in TCGA. Finally, we merged 35 samples derived from several normal tissues adjacent to cancerous tissue among the six TCGA disease projects(BRCA, GBM, KIRC, COAD, LUSC, and CESC). Preprocessing as mentioned above was also performed on the 35 normal controls. Although we simply integrate healthy samples from different tissues as a control set, the normalization step can remove differences between different tissues, and ensure the separability between cancer samples and healthy controls.

### 2.3. Methods

#### 2.3.1. SNF

Similarity network fusion(SNF) is a novel algorithm which integrates different omics data through computing and fusing patient similarity networks. SNF conduct the similarity fusing by iteratively updating every similarity network, making it more similar to the others with every iteration as follows:

$$P^{\left(v\right)}=S^{\left(v\right)}\times \left(\frac{\sum_{k\neq v}P^{\left(k\right)}}{m-1}\right)\times {\left(S^{\left(v\right)}\right)}^{T},v=1,2,\ldots ,m$$

where *P* represent the similarity matrix derived from each datasets, *S* represent the local affinity which only contains the nearest neighbors' information, and *m* is the number of different data types. Actually the iteration process means updating the similarity between node *i* and node *j* in *P*^(*v*)^ as the weighted sum of similarities between the K nearest neighbors of node *i* and those of node *j*. While neighbors' similarities are derived from the other *m* − 1 datasets.

The main contribution of SNF is it can solve the discrepancy of dimensions and variances in different omics datasets which may be the biggest challenge for omics data integration. And it has been widely used in many practical biological tasks. However, it still exists some limitations in this algorithm. (1) This procedure treats each network as the same without weights constraints. (2) There is only one connection path between different datasets that across two intermediate nodes which is insufficient for depicting complex network interaction. (3) The information exchange only exists in one dataset and the average of the others. There are no direct mutual adjustments between different datasets which may cover some interconnection between specific data types. The incomplete network connection model makes it difficult to recover the most precise global similarity pattern or resist high-level noise in biological data.

#### 2.3.2. Similarity Fusion Through High-Order Path

**Figure 1:**
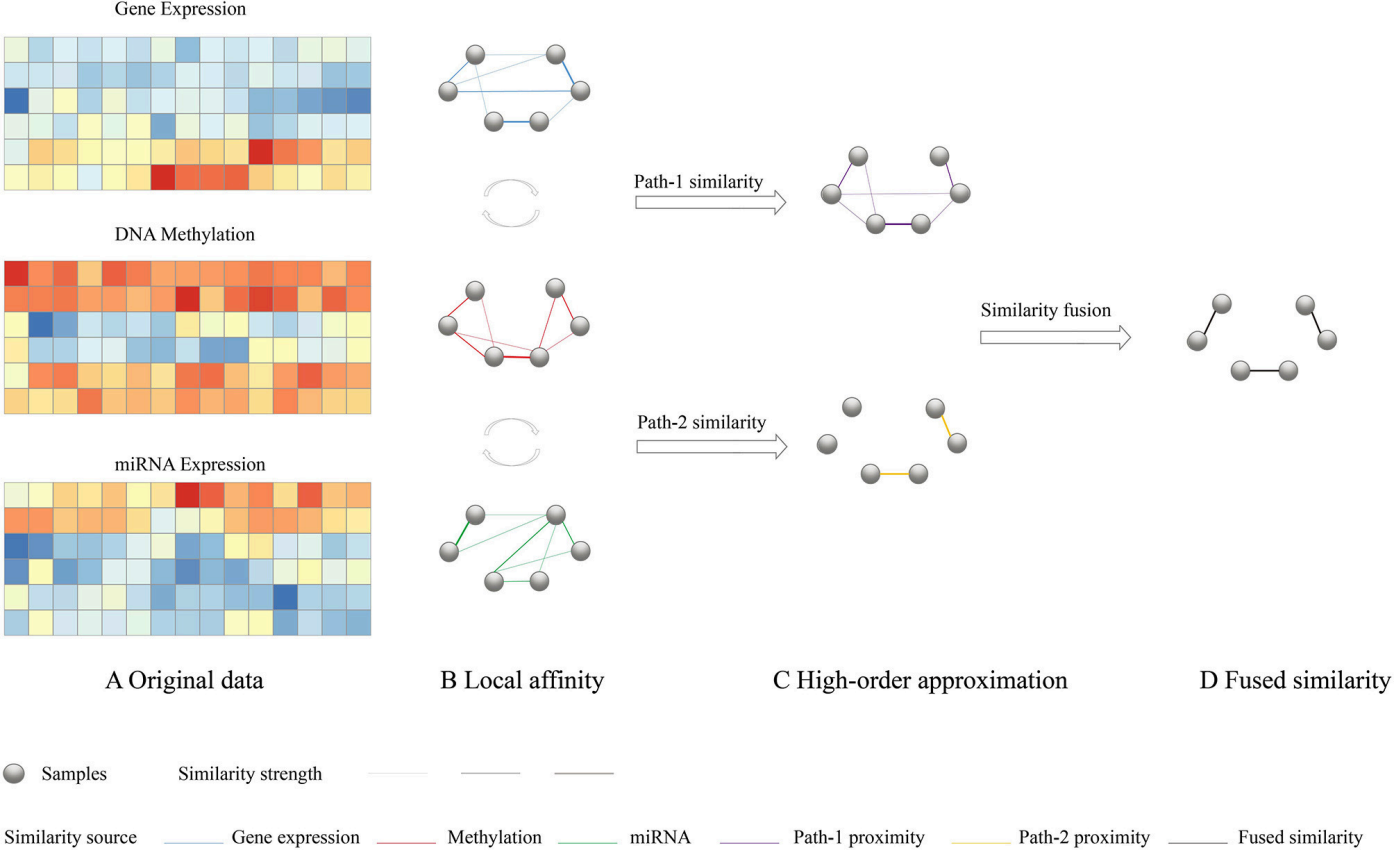
Overview of HOPES. **(A)** Illustrative example of input multi-omics data, including gene expression, DNA methylation, and miRNA expression data sets for the same sample cohort. **(B)** Local affinity of each data type was defined as part of the global similarity matrix that only contains edges among *K* nearest neighbors (*K* = 2). **(C)** Simplified illustration of path-1 elucidated similarity. We used matrix multiplication to transform the directed distance between samples to weighted one-hop distance. The purple edges represent correlations shared by two data types. **(D)** A path-2 elucidated similarity, in which only those edges with large similarity are preserved. Such edges are highlighted in yellow.

To have a consistent and highly representative global similarity, HOPES simulate three different network connection models with different path length and try to find the fused pattern which retains the maximal commonality. As it was depicted in Figure 1, (1) Path-0 similarity preserves the characteristics of each local affinity obtained using *K* nearest neighbor, (2) Path-1 similarity import one intermediate node to enhance the effect of each local affinity, (3) Path-2 similarity import two intermediate nodes to integrate interaction between different local affinity to enhance the commonality. The detailed numerical expression and constraint of the different order paths are as follows.

Suppose we have *C* different omics datasets, and their local affinities *S*_*i*_(*i* ∈ 1, …, *C*) were evaluated by a scaled exponential similarity kernel (Wang et al., 2014) see details in Supplementary Methods. First, for the path-0 similarity, the fused similarity is required to be close to each underlying affinity which can be simply characterized by minimizing average losses as follows:

(1) $$\underset{W}{\min}\sum_{i=1}^{C}\|W\;\cdot \;\Omega_{i}-S_{i}{\|}_{F}^{2}$$

where *W* is a *n* × *n* fused similarity matrix, *S*_*i*_ is local affinity extracted from *i*-th omics data, and Ω_*i*_ is a *n* × *n* matrix whose entries denote whether corresponding entries in *S*_*i*_ are equal to 0. There are *C* types of omics data.

Different from the path-0 similarity, we further propose path-1 similarity to retain the maximal commonality when filtering through each underlying affinity. Thus we assume the fused global similarity to be close to every one step transformed similarity by multiple each local affinity.

(2) $$\underset{W}{\min}\sum_{i=1}^{C}\|W-S_{i}W{\|}_{F}^{2}$$

It can be noted that ${\left(S_{i}W\right)}_{\left(m,n\right)}=\sum S_{i}(m,k)W(k,n)$ can be interpreted as the weighted sum of distance between the K nearest neighbors of node *m* and node *n* while neighbors' information are from dataset *i*, which represents W filtered by *S*_*i*_. Therefore, the aim of Equation (2) is to ensure proximity between the global affinity and the transformed affinity after it has been weighted by each local affinity. One can impose a stricter requirement that the fused global similarity is closed to the transformed similarity which has been filtered by each underlying local affinity through higher-order paths. For example, with path-2 proximity,

(3) $$\underset{W}{\min}\sum_{i=1}^{C}\sum_{j=1}^{C}\|W-S_{i}WS_{j}^{T}{\|}_{F}^{2}$$

Where ${\left(S_{i}WS_{j}\right)}_{\left(m,n\right)}=\sum S_{i}(m,k)W(k,l)S_{j}(l,n)$, It also represents the weighted sum of the distance between the K nearest neighbors of node *m* and those of node *n*, while neighbors' information of two vertexes is from two different datasets. This interactivity between different local affinity sharply strengthens the commonality requirement. The filtration process is supposed to weaken the original edges in W unless the correlation between node i and j is simultaneously supported by each pair of data types.

Finally, combining the aforementioned constraints for modeling proximities of various path orders, we propose the determination of the global affinity by minimizing the following energy function:

(4) $$\underset{W}{\min}\sum_{i=1}^{C}\left(\right\|\;W\;\cdot \;\Omega_{i}-S_{i}{\|}_{F}^{2}+\alpha \|W-S_{i}W{\|}_{F}^{2}+\beta \sum_{j=1}^{C}\|W-S_{i}WS_{j}^{T}{\|}_{F}^{2})$$

where α and β are hyperparameters that adjust the weight of different order constraints and can be empirically set. Details on parameter tuning was attached in the Supplementary Methods. The optimization problem can be solved through a consensus alternating direction minimization method (ADMM)(see Supplementary Methods for detailed solution procedure).

In conclusion, the three different order paths represent an incremental relationship from specificity to commonality and from weak constraint to strong constraint. They can simulate much more complex network connection models and set increasing consistency requirements on the global similarity. Therefore, we can take all the specialty of every single dataset, the interconnection between datasets, and global consistency into consideration and construct a more comprehensive and robust global similarity network. Moreover, the weights can be adjusted manually based on the real world condition which makes HOPES more flexible.

#### 2.3.3. Downstream Applications

Once we have the fused global similarity matrix, it can be the fundamental structure for much downstream analysis. The most directly is applying the spectral clustering to cluster the samples into different subgroups which can be used for cancer diagnosis or molecular subtyping. In this paper, to eliminate the variations due to clustering initialization, the consensus clustering (Monti et al., 2003) was used to enhance the reliability performance. It records the consensus across multiple clustering repeated trials based on one certain global similarity matrix to assess the stability of the clustering results.

Except for clustering, we also tried to project the global structure into specific characteristics in every single dataset. Since these features are the most relevant to the fused results, they can not only be prognostic valuable but also may indicate some interconnection between different omics layers. We located these features using MCFS, an unsupervised feature selection algorithm for multi-cluster data (Cai et al., 2010). After providing our fused similarity matrix *W* and the original omics data as input, the feature selection task can be modeled as a *L*1−*regularized* regression problem that exports the sparse coefficient vectors of features. In this case, we can easily select a series of most relevant features(corresponding to the non-zero coefficients).

## 3. Results

We designed a series of experiments to demonstrate the progress of HOPES by comparing it with four representative methods belong to three kinds of popular integration framework: network fusion-based SNF (Wang et al., 2014), joint latent variables-based iCluster+ (Mo et al., 2013), moCluster (Meng et al., 2015), and probabilistic model-based Clusternomics (Gabasova et al., 2017). Simulations and real data experiments were performed to evaluate the performance on global cluster structure detection and usability in clinical practice, respectively.

### 3.1. Experiments to Demonstrate the Accuracy and Robustness of HOPES With Simulated Data

To demonstrate the performance of HOPES in fusing multi-omics data, we first tested it on simulated datasets and compared it with SNF and moCluster. The simulated dataset was generated similarly to the one reported elsewhere (Shi et al., 2017). The simulated dataset was created to recapitulate the features of actual genomic data by combining biological variation levels from real data and a pre-defined cluster structure. The actual genomic profiles were downloaded from GEO (Barrett et al., 2013) with the following GEO codes: GSE51557, GSE73002 and GSE106453. These three were focused on DNA methylation (Conway et al., 2015), RNA expression (Nakagawa et al., 2008) and miRNA expression (Shimomura et al., 2016), respectively. Based on these actual genomic data we used the singular value decomposition (SVD) to fuse them with pre-defined cluster structure, and constructed two synthetic data sets (SimData1 and SimData2). SimData1 has a clear boundary between each cluster while SimData2 possesses fuzzy boundaries(see Supplementary Methods for more details).

**Figure 2:**
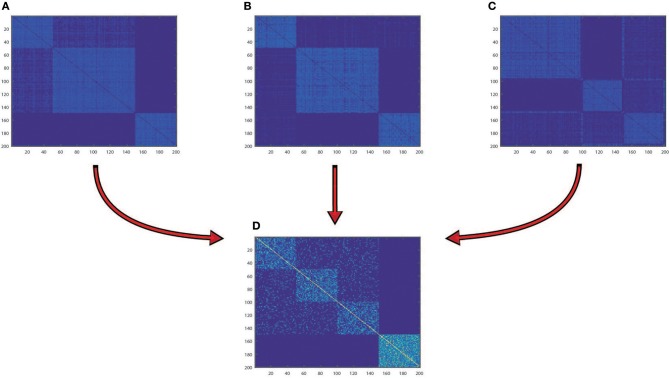
Synthetic data integration. Heatmaps of similarity matrix distinctly derived from RNA **(A)**, methylation **(B)**, miRNA data **(C)**, and fused data **(D)**, for which samples were arranged in the order of spectral clustering results.

We tested HOPES and the other methods on both simulation datasets under different levels of noise intensity to assess the information integration capability and robustness. We used the normalized mutual information (NMI) as a criterion for performance, and for each noise condition we ran repeated trials 20 times to eliminate accidental error. Collectively, all simulation results suggested that HOPES can always successfully recover the four pre-defined clusters from incomplete layers (Figure 2). As we demonstrate in data construction, the three single layers each contained an indivisible part. To dig out the real cluster information, an effective integration method was required. The proposed HOPES used the high order path distance among different data types to approximate the global similarity. The correlation information of nodes *i* and *j* will be weakened if it exists in only a single data layer, which ensures the separation of mixed groups in a single data source. Moreover, the progressive proximity model not only sets constraint on the high-order path distance, but also reconcile the extremely specific characteristics in each single data layer. Thus, it is promising for detection of the hidden cluster structure shaped by multi-source data.

**Figure 3:**
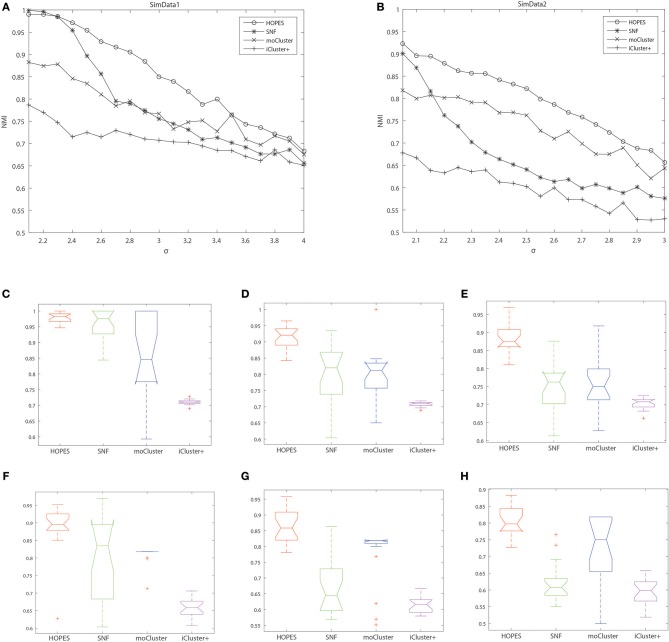
Cluster accuracy comparison between different methods on different simulation datasets. The upper panel represents the NMI of HOPES, SNF, and moCluster in SimData1 **(A)** and SimData2 **(B)** under incremental standard deviation of Gaussian noise. The lower panel shows the NMI boxplots in SimData1 **(C–E)** and SimData2 **(F–H)** among three methods under different noise levels (from left to right in the order of low, intermediate, and high), measuring their accuracy and stability on recovering the integrated pattern through partial layers.

The numerical results are shown in Table 1 and Figure 3, which suggest that HOPES outperformed the compared methods irrespective of the set signal and noise conditions, highlighted in bold in Table 1. It should be noted that Clusternomics show little tolerance on noise, because the lack of modeling for noise. For the rest three methods we can add the variance of Guassian noise to 3, while Clusternomics can only resist noise with variance lower than 1 (see Supplementary Figures for more details). In this section, we mainly discuss the performance on the rest four methods. It can be demonstrated that SNF achieved high precision when the noise level remained low; however, its robustness upon exposure to noise was insufficient. The low stability may be ascribed to SNF updating a fused network through a single local affinity and the other average similarity at every iteration. The update rule raises concern about the enhancement of erroneous information derived from one data layer, especially when edge points exist. However, HOPES provided path-2 elucidated similarity determined by each pair of data types which effectively solve it. In contrast, the latent variables-based methods such as iCluster+ and moCluster showed fairly good stability but poor accuracy for both of the synthetic datasets as noise increased. The iCluster+ modeled continuous variable as the linear combination of specific intercept term, common latent variables, and residual variance which all follow normal distribution. This assumption can fits our noise and original data setting, however, it can not accurately model the distribution of latent variables as a discrete sequence. So iCluster+ show good performance on dealing noises but unable to capture the global structures. The moCluster is based on a joint latent variable derived by consensus PCA, so it strongly relies on the selection of principal components. Moreover, the large gap between feature magnitude of distinct data types also affects the accuracy. More specifically, the boxplots indicate the degree of dispersion and skewness in the data, and show outliers during 20 repeated trials under low, medium, and high noise levels. As depicted in Figures 3C–H, HOPES achieved higher accuracy and more stable results within all three methods in SimData1. However, the results of moCluster were highly dispersed during repeated trials which makes the results less credible. After we imported edge points in SimData2, the discreteness of every method slightly increased, but HOPES still performed best, in accordance with the previous results. Interestingly, moCluster appears to be very stable when the noise level is low, but with moderate noise, almost half of the trials were quantified as outliers, which suggests this method exhibits large fluctuations.

**Table 1:** Performance measured by NMI on simulated datasets.

	**SimData1**			**SimData2**		
	**Low noise**	**Moderate noise**	**High noise**	**Low noise**	**Moderate noise**	**High noise**
HOPES	**0.972** ± **0.025**	**0.921** ± **0.044**	**0.858** ± **0.060**	**0.889** ± **0.056**	**0.838** ± **0.072**	**0.799** ± **0.071**
SNF	0.954 ± 0.061	0.811 ± 0.088	0.750 ± 0.075	0.822 ± 0.109	0.668 ± 0.095	0.619 ± 0.054
moCluster	0.864 ± 0.113	0.778 ± 0.088	0.748 ± 0.104	0.815 ± 0.015	0.786 ± 0.076	0.731 ± 0.108
iCluster+	0.710 ± 0.008	0.707 ± 0.008	0.693 ± 0.016	0.659 ± 0.026	0.617 ± 0.028	0.595 ± 0.036

### 3.2. Experiments for Cancer Diagnosis on Actual Cancer Datasets

**Figure 4:**
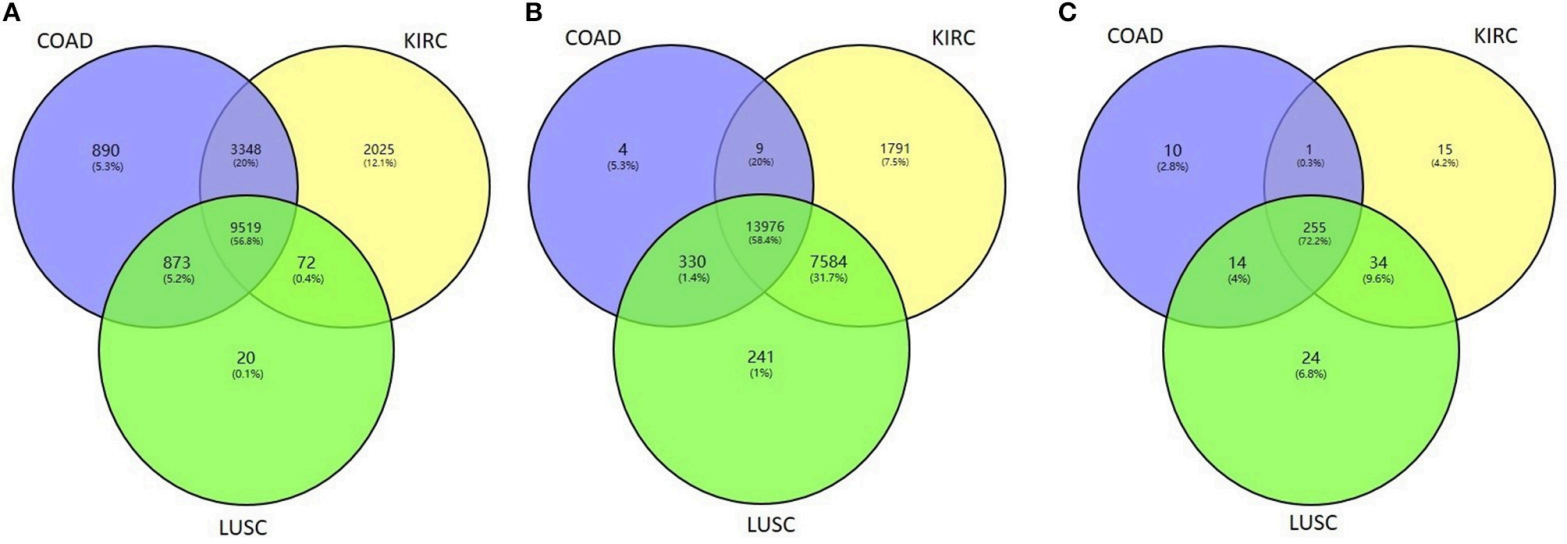
Venn diagram of the overlapping measurements among LUSC, COAD, and KIRC projects for the biological features of **(A)** gene expression, **(B)** methylation, and **(C)** miRNA expression.

We then tested whether the proposed method HOPES can distinguish tumor samples from normal controls based on their omics measurements. We applied the HOPES and other comparative methods to combinations of COAD (92 samples), KIRC (122 samples), LUSC (106 samples) and 35 normal controls. The gene expression, methylation, and miRNA expression data for these case/control sets and the overlap among them are shown in Figure 4. It can be noted that the amounts and proportion of common variables vary between different data types. The normal samples tested in this work were selected to have the matching characteristics. It can be noted that the amounts of variables vary from the expression of 280 miRNAs to 23,360 methylation sites, and miRNA measurements are shown to have the largest proportion of overlap among all of cancer types.

We calculated the classification accuracy on the collected tumor vs. normal samples. Table 2 shows the classification performance either by one single set of data or by the fused methods, in which the most highest accuracy were highlighted in bold. The results reflect that, at the single data level, miRNA with the smallest number of measurements showed the best performance regarding sample classification while methylation showed the worst performance. On average, the performance on fused data derived by HOPES and SNF is uniformly better than that for a single source. The good performance of data fusion is attributed to its capability of resisting erroneous correlations or even negative effects, which not only enhances accuracy but also generates more stable results.

**Table 2:** The accuracy for cancer diagnosis of different methods.

	**COAD**	**KIRC**	**LUSC**
Gene expression	0.8740	0.5159	0.8865
Methylation	0.4882	0.6433	0.6667
miRNA expression	0.8504	0.8471	0.8652
HOPES(fused)	**0.8976**	**0.9236**	**0.9286**
SNF(fused)	0.8976	0.9172	0.9078
iCluster+(fused)	0.6299	0.5923	0.6383
moCluster(fused)	0.7559	0.707	0.7801
Clusternomics(fused)	0.5276	0.6433	0.8865

**Figure 5:**
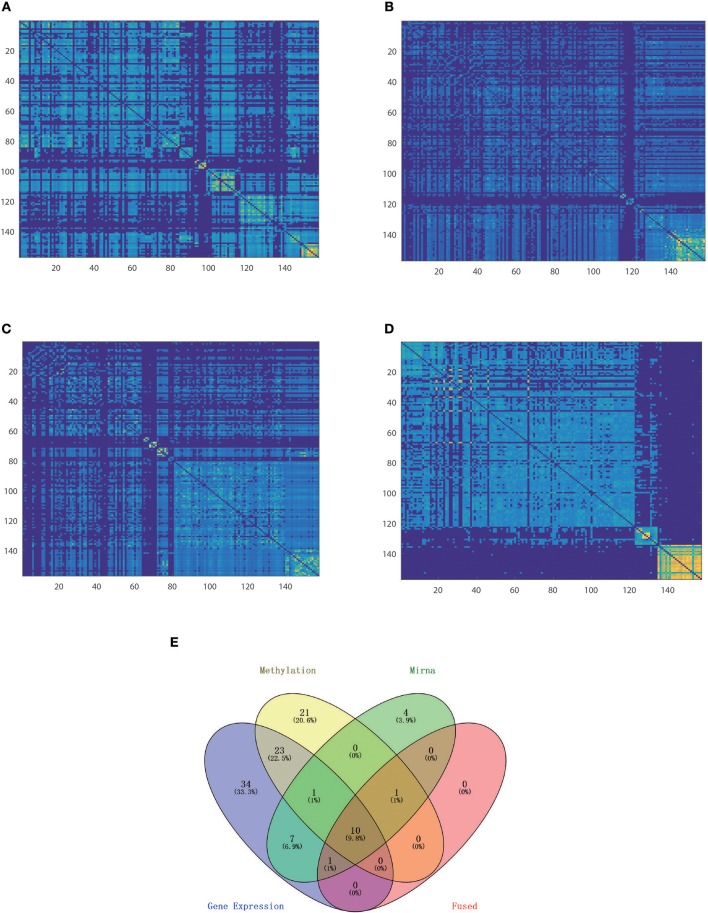
Comparison of classification performance based on single and fused data. Heatmap of similarity matrix derived from **(A)** gene expression data, **(B)** methylation data, **(C)** miRNA expression data, and **(D)** fused data where samples were gathered by classification results on the corresponding dataset. **(E)** Venn plot shows the distribution of mis-assigned specimens in all of the four data sets.

Nevertheless, integration methods such as iCluster+ which splices all of the features, strongly rely on a priori gene selection; therefore, if the number of variables is imbalanced, it will be difficult to retain positive information. Thus, the classification accuracy falls in between the worst and best of single level analyse, so as for moCluster. The Clusternomics extract the global assignment based on the mixture of local partitions, so if clustering results were obscure in single data layer the global performance can not be satisfied. The sample size also influence the performance of Clusternomics a lot. We take an example of KIRC dataset for further analysis. One can see that the fused data clustered tightly and uniformly, as shown by the heatmap of the similarity matrix (Figure 5). One can see that the clustering result by the proposed HOPES achieved superior performance (Figure 5D) to that by each single source (Figures 5A–C). In Figure 5D shows distinct boundaries between different clusters and uniform structure within each cluster. The fused similarity between healthy samples is far greater than cancer samples, which demonstrates the heterogeneity of cancer. We also created a Venn diagram to examine the sample assignment by each single source or by the fused one. We found that the fused data by HOPES are robust to mistakes in each single source. More precisely, for 65% (102 of 157) of samples, there were incorrect assignments in at least one single data type analysis, while for 33% (53 of 157) of cases, the classification results were wrong in at least two single data types. However, only 7.6% (12 of 157) of cases were mis-assigned by our method (Figure 5E).

### 3.3. Prognostic Performance on Actual Cancer Datasets

**Figure 6:**
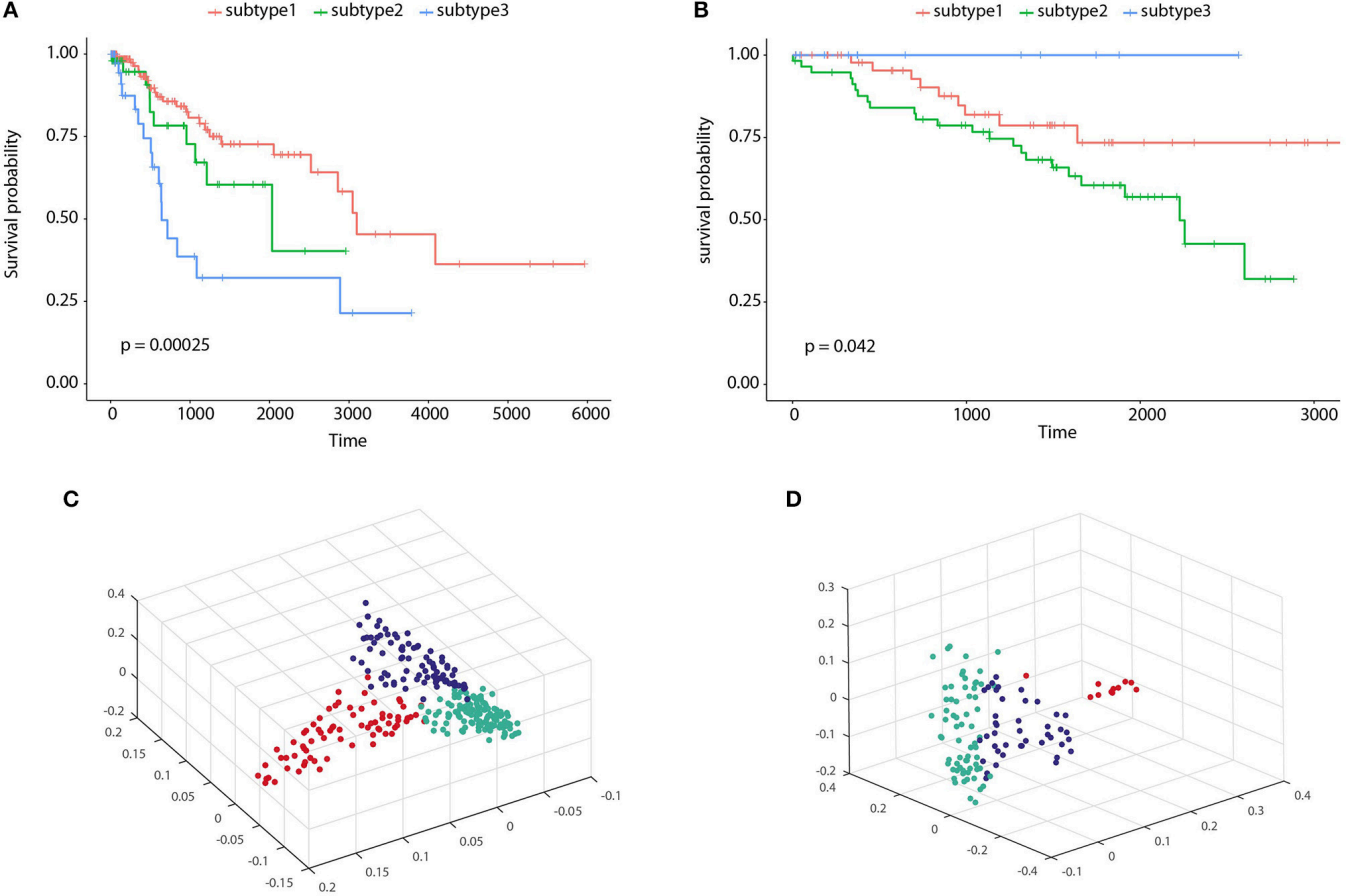
Clustering results of CESC and KIRC. The Kaplan-Meier survival curves *P*-values are recorded in Table 3 for **(A)** CESC and **(B)** KIRC, and 3D scatter plots for **(C)** CESC and **(D)** KIRC. Vertexes of scatter plots represent samples colored by their cluster label; the *x*-, *y*-, and *z*-axis represent the first three principal components of the fused data matrix.

To illustrate the prognostic ability of the elucidated similarity, we applied HOPES to five tumor omics datasets, namely CESC, GBM, COAD, KIRC, and LUSC. The similarities obtained by SNF and HOPES were used to cluster each tumor sample into three subtypes. Their corresponding survival curves were drawn and quantified by the log-rank test. The statistical significance of differences between them was denoted by the *P*-value. To facilitate visual comparisons, the results on both the survival curves and the first three principal components are shown in Figure 6 and Supplementary Figure 3. The survival curves resulting from HOPES can be observed to achieve the smallest *P*-value, highlighted in bold in Table 3. Consistent with the results in synetic experiments, HOPES show the most clinical significant and reliable performance in all datasets. Since COAD only contains 92 samples with more than 20,000 gene features, the Clusternomics can not fit a mixture model for COAD.

**Table 3:** Survival analysis by Log-rank test on five tumor datasets.

	**CESC**	**COAD**	**GBM**	**KIRC**	**LUSC**
HOPES	**0.000248**	0.00918	**0.000224**	**0.0417**	**0.00132**
SNF	0.000626	0.038	0.000621	0.124	0.00551
iCluster+	0.63	**0.00316**	0.751	0.206	0.0082
moCluster	0.0567	0.139	0.0207	0.0667	0.00193
Clusternomics	0.162	–	0.048	0.129	0.00504

**Figure 7:**
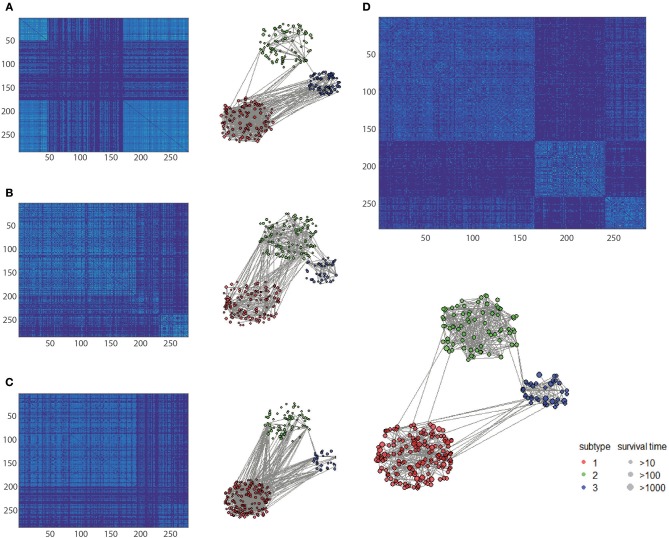
Patient similarity networks for 284 CESC samples of each single data type independently compared to the fused similarity network. Heatmaps on similarity for **(A)** gene expression, **(B)** methylation, **(C)** miRNA expression, and **(D)** integrated matrix are shown.

To clarify the beneficial characteristics of the similarity elucidated by HOPES, we took another example of CESC for further analysis. We compared the clustering results on each single type of omics data alone with those for the elucidated one. The results are plotted in a heatmap as shown in Figure 7. Notably, it is difficult to cluster each single type of omics data into sub-clusters. There are no legible block structures in Figure 7A, or only tiny sub-clusters in Figures 7B,C. Between different clusters, the cross section shows small differences in color, implying that the differences were negligible. In comparison, the clustering results after HOPES were shown to feature three distinct sub-clusters. The last sub-cluster in the bottom-right corner exhibits a fairly homogeneous color within the clusters. Moreover, we can deduce that there are two clusters, upon clustering by gene expression, as shown in Figure 7A. There are no obvious sub-clusters either by methylation level (Figure 7B) or by miRNA expression (Figure 7C). In comparison, the clustering results after HOPES were shown to feature three distinct sub-clusters. The last sub-cluster in the bottom-right corner exhibits a fairly homogeneous color within the clusters. The elucidated similarity makes it markedly easy to find sub-clusters that were concealed in the analyses for each type of omics data alone.

We also found that the elucidated similarity highlights the molecular heterogeneity in cervical carcinomas. The subtyping by HOPES differed depending on the histological classification, showing a discrepancy between phenotype and gene-level types. For instance, the sub-clusters by HOPES largely corresponded to those by methylation level. The CESC project classified samples into six subgroups by histology. To determine the correspondence between the histological classification and HOPES, we merged four different types of adenocarcinoma into one type, as used in studying cervical cancer (Cancer Genome Atlas Research Network et al., 2017). The clusters produced by HOPES strongly correlated with the histological types, but were not the same; our cluster 3 contained all of the adenosquamous cases, while cluster 2 mainly consisted of cervical squamous cell carcinoma samples. We used the χ^2^ test to determine whether the two clustering results are significantly associated, and our cluster results showed a strong correlation with each single genomic data cluster, with small *P*-values (gene expression *P* = 1.28 × 10^−6^; methylation *P* = 7.94 × 10^−9^; miRNA expression *P* = 2.2 × 10^−16^).

### 3.4. Functional Annotation of Relevant Features Among Cervical Cancer Subtypes

To demonstrate the biological significance of subtype derived by HOPES, we extracted the subset of the most relevant features among the original features and conducted a series of functional analyse on it. We chose the 15 most relevant features in gene expression, methylation, and miRNA data for further analysis.

**Figure 8:**
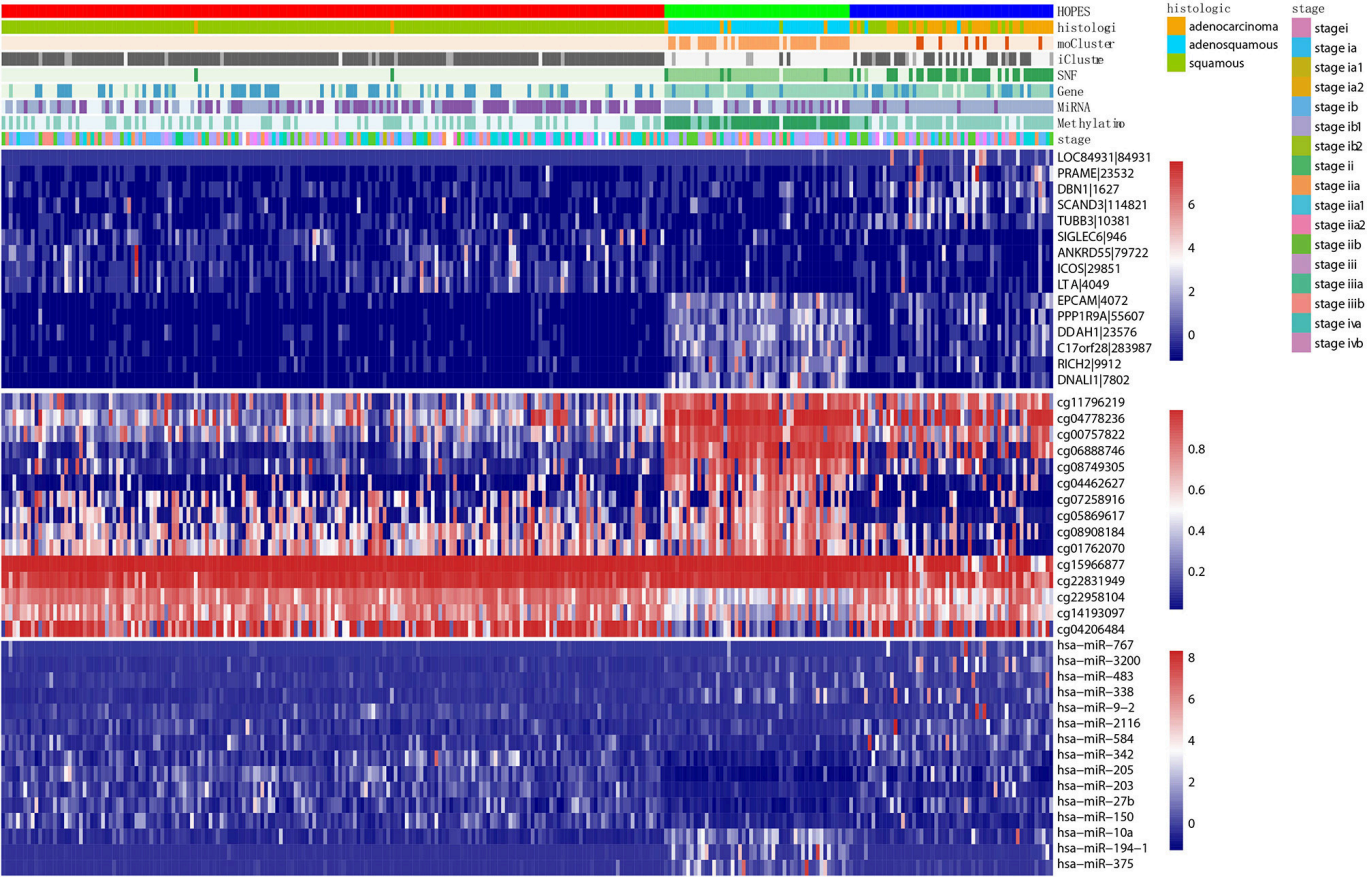
Multiplatform integrative clustering of cervical cancer. Clustering of 284 cervical cancer samples was performed based on different datasets (i.e., gene expression, methylation, miRNA, and fused data) using different methods(HOPES, SNF, iCluster+, moCluster). Moreover, the histological type and clinical stage of patients are also indicated in the legends. The heatmaps show the selected top 15 mRNAs, CpG sites, and miRNAs that are either significantly associated with HOPES groups or have been identified as markers in previous analyses; different datasets correspond to distinct color scale bars.

First, we constructed a corresponding heatmap with different clustering labels, In Figure 8, selected signatures of all three data types are merged, showing a clear block form corresponding to the HOPES subgroup. As long as these selected features are differentially expressed following our clustering result, their biological annotation can help us to confirm that the separation created by HOPES is not only clinical meaningful but also biologically significant. In terms of the gene expression pattern, subtype 1 (red), corresponding to lower expression in EPCAM, PPP1R9A, DDAH1, C17orf28, RICH2, and DNALI1, showed a longer survival time, while subtype 2 exhibited completely the opposite performance in the same gene set. The subgroup with the poorest prognosis (blue) significantly corresponded to LOC84931, PRAME, DBN1, SCAND3, and TUBB3 over-expression. The methylation data specifically highlight subgroup 1 in the first five CpG sites(cg11796219, cg04778236, cg00757822, cg06888746, cg08749305); subgroup 2 shows down-regulation in the last three CpG sites (cg22958104, cg14193097, cg04206484); while subgroup 3 is relatively down-regulated in cg07258916, cg05869617, cg15966877, and cg22831949. The heatmap of miRNA shows increased expression of hsa-miR-767, hsa-miR3200, and hsa-miR-483, which correlates with decreased survival probability and clearly up-regulated expression of hsa-miR-10a, hsa-miR-194-1, and hsa-miR-375 in subgroup 2.

**Figure 9:**
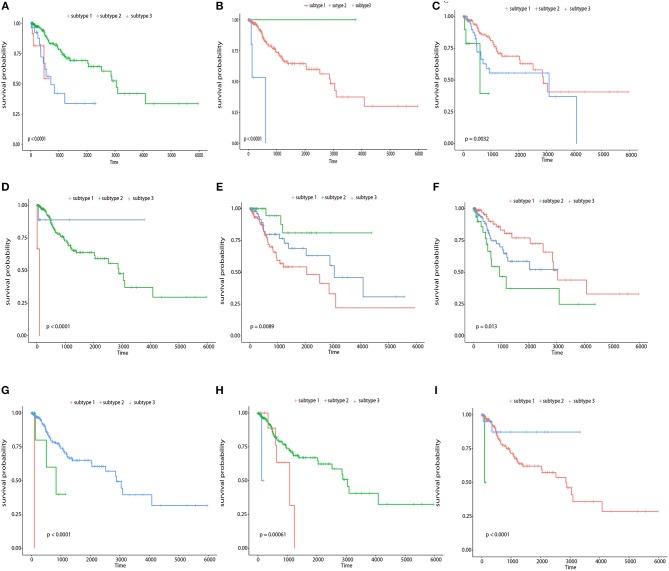
Survival curves of top 3 most significant features in genes, CpG sites, and miRNAs respectively The Kaplan-Meier survival curves for the top 3 most significant genes, namely, LOC84931, TUBB3, and DBN1 **(A–C)**, the top 3 most significant CpG sites, namely, cg1596687, cg07258916, and cg11796219 **(D–F)**, and the top 3 most significant miRNAs, namely, hsa-miR-767, hsa-miR-483, and hsa-miR-9-2 **(G–I)**.

Second, we performed survival analysis on each single feature using the kmeans as a general clustering method, and found that more than 1/3 relevant features showed good partition ability with a Log-rank test *p*-value< 0.05 including five genes (LOC84931, DBN1, SCAND3, TUBB3, ICOS), six CpG sites (cg11796219, cg08749305, cg07258916, cg05869617, cg01762070, cg15966877), and six miRNAs (hsa-miR-767, hsa-miR-3200, hsa-miR-483, hsa-miR-9-2, hsa-miR-584, hsa-miR-342). Figure 9 shows the Kaplan-Meier survival curves of the top 3 most significant features in genes, CpG sites, and miRNAs. Among these genes, DBN1 was detected as a useful oncofetal biomarker (Iyama et al., 2016). It is involved in migration and invasion of glioma, colon, bladder and lung cancer (Mitra et al., 2011; Terakawa et al., 2013; Lin et al., 2014; Zwiener et al., 2014; Xu et al., 2015); TUBB3 was assessed as one of the predictive and prognostic factors in cervical cancer patients under different neoadjuvant regimens (Zwenger et al., 2015). It was also defined to be a useful prognostic biomarker in patients with advanced NSCLS (Li Z. et al., 2014). Moreover, ICOS was also included in one of the genotype combinations (CD28/IFNG/ICOS) that is associated with cervical cancer (Guzman et al., 2008). In analyzing each single CpG site, an R package, “IlluminaHumanMethylation450kanno.ilmn12.hg19” was applied to match each CpG site with reference gene region. The most significant features, included cg22831949, falls in PTPRN2, which was found to inhibit apoptosis and promote cancer formation in breast cancer (Sorokin et al., 2015); cg07258916 corresponding to PLXNA4 which belongs to the plexin family, and was previously indicated to inhibit tumor cell migration (Balakrishnan et al., 2009); cg11796219 matched with C3orf21, while C3orf21 ablation was proved to promote cell proliferation, inhibite apoptosis and accelerate cell migration in lung cancer. Selected miR-767 contributes to the decrease of TET activity, which is a hallmark of cancer (Loriot et al., 2014). It also known as risky miRNA that significantly correlates with clinical outcomes in GBM (Li R. et al., 2014). Moreover, miR-483 can play the role of an antiapoptotic oncogene in many human cancers, such as Wilms' tumors, colon, liver, and breast cancers (Veronese et al., 2010). It was also identified as predictors of poor prognosis in adrenocortical Cancer (Soon et al., 2009). miR-9 was proved to be correlated with MYCN amplification, tumor grade, and metastatic status (Ma et al., 2010), more specifically, it was found to be associated with clear cell renal cell carcinoma, breast cancer, gastric carcinoma, and brain tumors (Lehmann et al., 2008; Luo et al., 2009; Nass et al., 2009; Hildebrandt et al., 2010).

**Figure 10:**
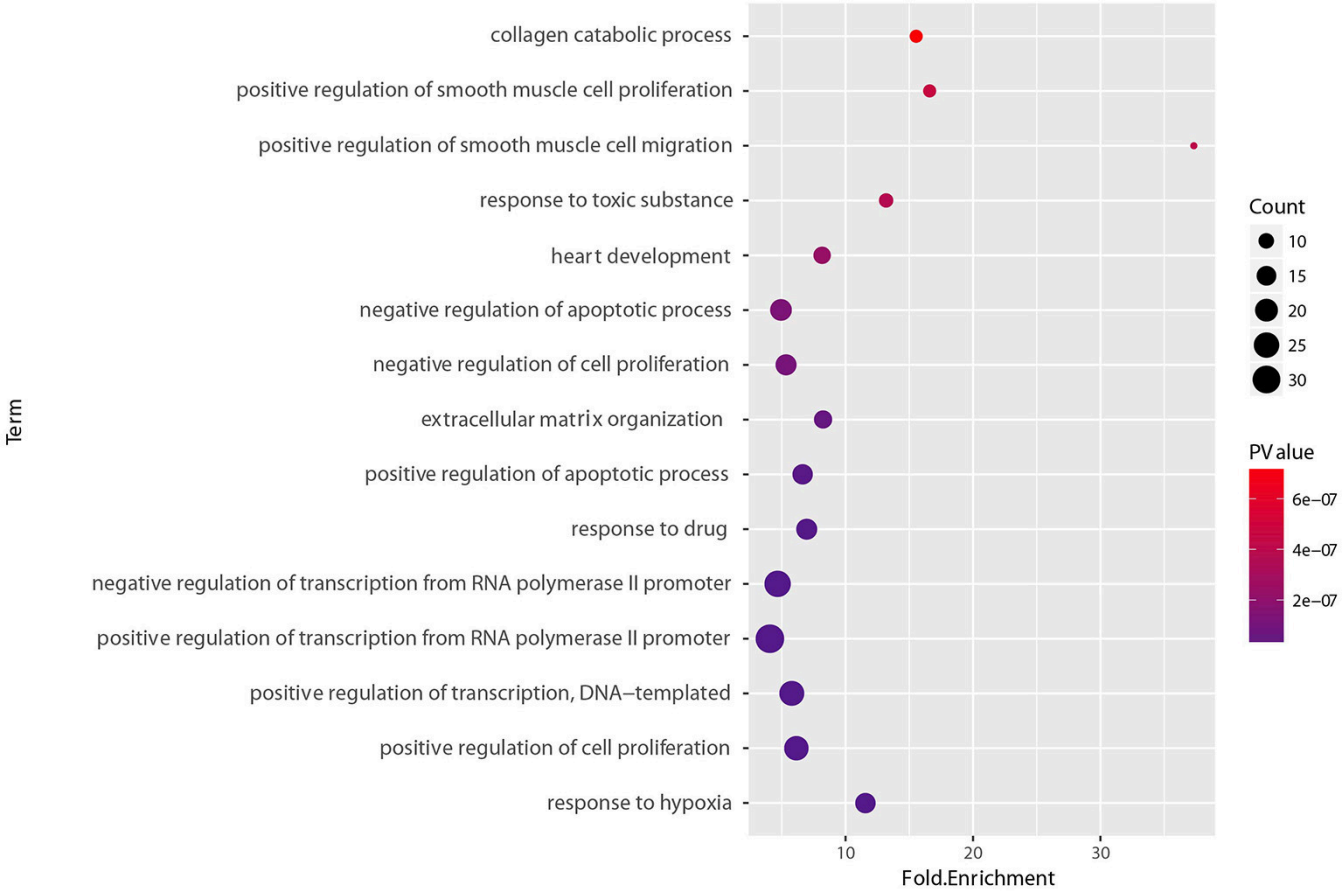
GO biological process enrichment analyses of set of 173 core genes gene count, *p*-value, and fold enrichment of GO biological process terms with *P*-value< 10^−6^.

**Figure 11:**
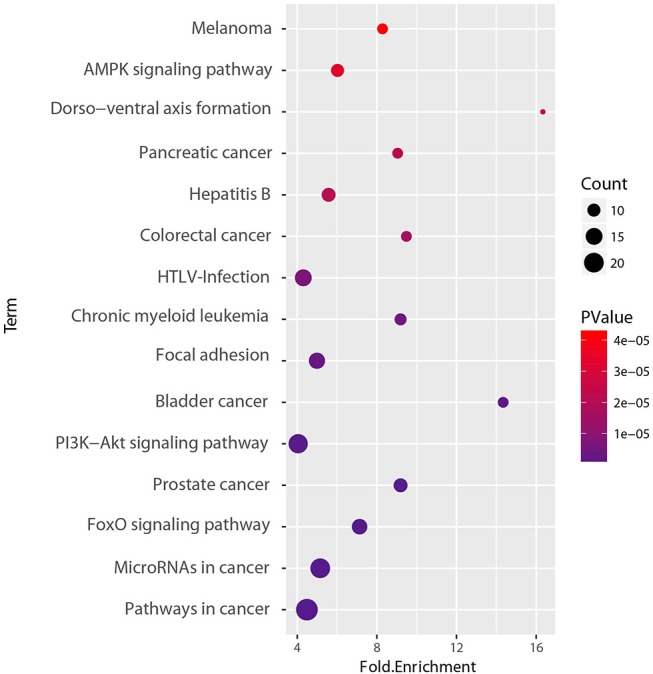
KEGG pathway enrichment analyses of set of 173 core genes gene count, *p*-value, and fold enrichment of KEGG pathways with *P*-value< 10^−4^.

To determine the functional relevance of the selected features, the identified genes, target genes of CpG sites and miRNAs were merged as a core set. We then performed the GO enrichment analysis (Ashburner et al., 2000) and KEGG pathway analysis (Kanehisa et al., 2011) on it using DAVID tools (Huang et al., 2008, 2009). The genes targeted by miRNAs were predicted by miRTarBase, an experimentally validated miRNA-target interaction database (Chou et al., 2017). We only used the interactions supported by strong experimental evidence (reporter assay or western blot). Finally, the core gene set included 173 genes consisting of 15 original genes, 15 methylation related genes, and 143 miRNA targets. We found that the whole core gene set was enriched in 56 GO biological process terms, with Benjamini-corrected *p*-value < 0.05. Figure 10 depicts GO terms with *p*-value < 10^−6^, notably, these significant terms strongly correlate with cancer. An example of this is the most significant term, namely respond to hypoxia. Numerous research has confirmed that pathological hypoxia plays a pivotal role in cancer progression and migration (Muz et al., 2015). In addition, the Hypoxia-inducible factor 1α, which regulates genes involved in response to hypoxia was proved as a strong prognostic marker in early stage cervical cancer (Birner et al., 2000). The regulation of cell proliferation, regulation of transcription from RNA polymerase II promoter, and regulation of apoptotic process participate in the full life-cycle of tumors (Takeshima et al., 2009; Vander Heiden et al., 2009; Wong, 2011). For KEGG analysis, a total of 46 pathways (Benjamini-corrected *p*-value < 0.05) were identified, Figure 11 shows pathways with *p*-value < 10^−4^. Among these pathways, cancer was the most common subclass such as pathways in cancer, microRNAs in cancer, Bladder cancer, colorectal cancer and pancreatic cancer. Besides direct cancer pathways, the PI3K-AKT-FoxO signaling cascade was identified, which has been previously identified to be involved in cancer and aging (Zhang et al., 2011). The PI3K/Akt signaling pathway leads to the inhibition of the downstream targets FoXO transcription factors, while FoXO is associated with cell cycle progression (Medema et al., 2000), apoptosis (Urbich et al., 2005), and angiogenesis (Tang and Lasky, 2003). There is another research revealed that the activation of AMPK impedes cervical cancer cell growth through this PI3K-AKT-FoxO axis (Yung et al., 2013).

In conclusion, we performed survival analysis, GO enrichment analysis, and KEGG pathway analysis on a subset of the most relevant features of gene expression, methylation and miRNAs corresponding to our HOPES subgroups. We found that these selected features were of great significance in cancer clinical outcomes and biological function such as cancer cell proliferation, apoptosis, and angiogenesis. These findings not only demonstrate the biological meaning of our integrated clustering results, but also indicate that HOPES can act as the anterior work for prognostic biomarker detection.

## 4. Discussion

The integrated analysis of multi-omics data can facilitate the study of molecular events at different periods of cancer progression and development, and complementary information can remove the effect of noise, leading to precise and useful classification results. Our proposed HOPES method integrates the similarity of different data layers to overcome the dimension and scale heterogeneity that hinders latent variable-based methods. The progressive fusion model based on high-order path similarity can evaluate the strength of single data level specificity and global level consistency together for a consistent and highly representative global similarity. The derived global similarity can filter erroneous or single level specific ties. This procedure can solve the issue of inducing too much noise or distortions by partial structures in a single data set, when we integrate all of the similarity information from each data type. Downstream consensus spectral clustering contributes to the obtainment of reliable clustering results.

In practice, our method shows superior capabilities in distinguishing global patterns through multiple source data. In addition, HOPES show great robustness compared to the other methods which are constrained by sample size or priori feature selection. Since HOPES only used the sample similarity information, its performance is independent of the data source, so it is promising for general usage. The fused similarity matrix shows the higher accuracy of tumor classification than any single data type or other integration methods. Moreover, the clustering results of cancer patients feature significant separation regarding a prognostic indicator (survival time), which can contribute to cancer subtyping at the molecular level and further clinical treatment. The obtained subgroups are also shown to be promising for the identification of potential biomarkers by revealing the key components that drive the differences between subgroups. The enrichment analysis on the key components confirmed the power of HOPES in discriminating the biomarkers.

## Data Availability

The CESC dataset generated during and analyzed during the current study are available in the Broad Institute TCGA Genome Data Analysis Center with identifier “https://doi.org/10.7908/C11G0KM9” (Broad Institute TCGA Genome Data Analysis Center, 2016). The BRCA, LUSC, COAD, and GBM datasets that support the findings of this study are provided by Wang et al. (2014). The Code used in this publication is freely available at github.com/scutbioinformatics/HOPES.

## Author Contributions

AX and HC conceived, designed, and supervised all phases of the project. AX performed experiments and wrote the manuscript. AX and JC performed the bioinformatics analysis. JC, HP, and GH contributed to discussions, and editing of the paper. All authors read and approved the final manuscript.

### Conflict of Interest Statement

The authors declare that the research was conducted in the absence of any commercial or financial relationships that could be construed as a potential conflict of interest.
