# Supplementary material for: Simultaneous Interrogation of Cancer Omics to Identify Subtypes With Significant Clinical Differences
Source: Front Genet. 2019 Mar 28;10:236. doi: 10.3389/fgene.2019.00236 (PMC6448130; doi:10.3389/fgene.2019.00236)
Supplement: Supplementary file 1 [file Data_Sheet_1.PDF]

frontiers

Supplementary Material: Simultaneous interrogation of cancer omics to identify subtypes with significant clinically differences

1 SUPPLEMENTARY METHODS

1.1 Constructing local affinity matrix

Suppose we have n samples and m measurements at a particular omics level, for example, DNA methylation. The local affinity matrix for the observed sample at this level is represented by an $n \times n$ adjacency matrix W. The similarity for the i-th and j-th sample is denoted by $W(i, j)$, defined by a scaled exponential similarity kernel:

$$W(i,j) = exp\left(-\frac{d^2(x_i, x_j)}{\rho \varepsilon_{i,j}}\right) \tag{S1}$$

where $d(x_i, x_j)$ is the Euclidean distance for sample i and j. The parameter ρ is a scaling constant used to control the exponential decay speed. The constant $\varepsilon_{i,j}$ is used to correct the measurement bias, defined by

$$\varepsilon_{i,j} = \frac{\mu\left(d(x_i, N_i)\right) + \mu\left(d(x_j, N_j)\right) + d(x_i, x_j)}{3}$$

where $\mu\left(d(x_i, N_i)\right)$ is the mean of the distances between x_i and each of its neighbors.

The similarity matrix is further normalized by $W = D^{-1}W$ to achieve uniform measurement among different omics types. Here, D is a diagonal matrix whose diagonal entry is, $D(i,i) = \sum_j W(i,j)$, so that $\sum_j W(i,j) = 1$. Finally, the local affinity matrix is computed by:

$$S(i,j) = \begin{cases} \dfrac{W(i,j)}{\sum_{k \in N_i} W(i,k)} & j \in N_i \\ 0 & otherwise \end{cases} \tag{S2}$$

1.2 Numerical scheme to find the elucidated similarity

We propose the determination of the fused global affinity by minimizing the following energy function:

$$\min_W \sum_{i=1}^{C} (\|W \cdot \Omega_i - S_i\|_F^2 + \alpha \|W - S_i W\|_F^2 + \beta \sum_{j=1}^{C} \|W - S_i W S_j^T\|_F^2) \tag{S3}$$

We solved the problem in Eq (S3) by using a consensus alternating direction minimization method (ADMM). By introducing variables $W_1, W_2, \cdots, W_C$, the optimization problem could be rewritten as:

$$\min_{W_1,\dots W_C,W} \sum_{i=1}^{C} \|W_i \cdot \Omega_i - S_i\|_F^2 + \alpha \sum_{i=1}^{C} \|W_i - S_i W\|_F^2 + \beta \sum_{i=1}^{C}\sum_{j=1}^{C} \|W_i - S_i W S_j^T\|_F^2 \tag{S4}$$

subject to

$$W_i = W, i = 1, ...C$$

After this modification, the decomposable nature of ADMM makes it easy to solve the problem by minimizing C subproblems followed by average updates. In particular, the i-th subproblem in the t-th iteration is:

$$\begin{aligned} W_i^{(t)} = & \underset{W_i}{\operatorname{argmin}} \quad \|W_i \cdot \Omega_i - S_i\|_F^2 + \alpha \|W_i - S_i W^{(t-1)}\|_F^2 \\ & + \beta \sum_{j=1}^{C} \|W_i - S_i W^{(t-1)} S_j^T\|_F^2 \\ & + \frac{\rho}{2} \|W_i - W^{(t-1)} + \omega_i^{(t-1)}\|_2^2 \\ \omega_i^{(t)} = & \omega_i^{(t-1)} + W_i^{(t)} - W^{(t-1)} \end{aligned} \tag{S5}$$

where ρ is the Lagrange coefficient. After each round, when one has $W_i^{(t-1)}$ for $i = 1, 2, \cdots, C$, we can update $W^{(t)}$ by

$$W^{(t)} = \frac{1}{C} \sum_{i=1}^{C} W_i^{(t-1)} \tag{S6}$$

Each minimization subproblem is convex and differentiable. Thus it has an explicit solution of

$$\begin{aligned} W_i^{(t)} = & (2S_i \cdot \Omega_i + 2\alpha S_i W^{(t-1)} + 2\beta \sum_{i=1}^{C} S_i W^{(t-1)} S_j^T + \\ & \rho W^{(t-1)} - \rho \omega_i^{(t-1)}) ./ (2\Omega_i \cdot \Omega_i + (2\alpha + 2\beta C + \rho) I) \end{aligned} \tag{S7}$$

where I is a $n \times n$ all-one matrix with each entry being 1. The aforementioned solution iteratively solves each subproblem until convergence.

1.3 Synthetic data construction

A sample process of creating the simulated data is depicted in Supplement Figure S1. We first have the actual genomic data from RNA expression, DNA methylation, and miRNA expression in the first column. Then we create three distinct matrices with various clusters in the second column. The simulated data were obtained by the combination of the first and the second column, respectively.

The actual genomic profiles involved researches on DNA methylation, RNA expression and miRNA expression were downloaded from GEO (https://www.ncbi.nlm.nih.gov/geo/) with the following GEO codes: GSE51557, GSE73002 and GSE106453. For each data type, a missing-value imputation was performed to preprocess the data. 200 samples with a missing rate lower than 20% were randomly

selected. For those having missing-value data, a K-nearest neighbor (KNN) imputation scheme was used to complement it by filling the empty area with the mean value of non-empty neighbors from the 200 complete samples.

After preprocessing we obtained three actual genomic data matrices, referred to as X_1, X_2, and X_3, where rows represent biological features and columns represent samples. The measurement information as well as the sample variations for the three genomic data could be derived by singular value decomposition (SVD) to each matrix.

$$X_i = U_i D_i V_i^T \tag{S8}$$

U_i and V_i were preserved to provide sample and measurement characteristics of data, respectively. Now, we can construct three matrices with each consisting of multiple clusters. The three matrices were also contaminated by standardized white noise and denoted by $\hat{X}_1, \hat{X}_2$ and $\hat{X}_3$. They are constructed as:

$$\hat{X}_i = B_i + \varepsilon \tag{S9}$$

where $\varepsilon \sim N(0, 1)$ represents random bias. The matrix B_i represents the pseudo-biological expression level with various clusters. In our example, it is obtained by randomly sampled from $\{0, 2, 4, 6\}$ without repetition within fixed position. For instance, samples 1-50, 51-150, and 151-200 in B_1 with $\mu_{k(k=1,2,3)} = \{0, 2, 6\}$; samples 1-50/101-150, 51-100, and 151-200 in B_2 with $\mu_{k(k=1,2,3)} = \{0, 4, 6\}$; samples 1-100, 101-150, and 151-200 in B_3 with $\mu_{k(k=1,2,3)} = \{4, 2, 6\}$. Clearly the dataset consists of distinct clusters such that any two of them can not jigsaw a complete map. Similarly, the three matrices were factorized by SVD as

$$\hat{X}_i = \hat{U}_i \hat{D}_i \hat{V}_i^T \tag{S10}$$

Finally, three simulated datasets with biological knowledge and cluster structures were combined together as:

$$X_{Sim_i} = U_i D_i \hat{V}_i^T \tag{S11}$$

In order to test the performance of HOPES in radical scenario, we modified the SimData1 by artificially blurring the boundaries among each cluster. We named the modified data as SimData2. The modification process started from sampling around 10%-20% of samples from each cluster. The samples were changed to be similar to the ones in other different clusters, resulting to the assignment of these samples being difficult. For instance, samples 1-40, 61-150, and 161-200 with $\mu_k = \{0, 2, 6\}$, while samples 41-50, 51-60,151-160 with $\mu'_k = \{0.8, 1.2, 4.8\}$.

To make the clustering more challenging, the Gaussian noise ($N(0, \sigma)$) with zero mean value and different standard deviation σ is added to the both SimData1 and SimData2. For SimData1, we set the noise intensity to increase from 2 to 4 at intervals of 0.1 (corresponding to the signal-to-noise ratio decreasing from -1 to -9.5). In addition, we increased the noise intensity from 2 to 3 at intervals of 0.05 (corresponding to the signal-to-noise ratio decrease from -1 to -5.9) for SimData2. Our signal-to-noise ratio was clearly negative, which approached the actual genomic data in our view. Unambiguous instinct characteristics were, quiet reasonably, buried by noise with several times the intensity of them. We further define three levels of noise contamination, low, mediate, and high, with $\sigma = (2.4, 2.7, 3)$ for SimData1, and $\sigma = (2.15, 2.35, 2.55)$ for SimData2.

1.4 Parameter tuning

HOPES model contains two parameters (α, β) which control the strength of constraints derived by different order path similarity. We use the simulation experiments to select paired parameters with best performance. We choose [0,2] and [0,2] as the range of parameters α and β to conduct 20 repeated trials on SimData1 with moderate noise level for each paired parameters, and use the NMI to evaluate the performance(Supplement Figure S2).

2 SUPPLEMENTARY FIGURES

2.1 Supplementary Figure 1

Demonstration of constructing simulated data.

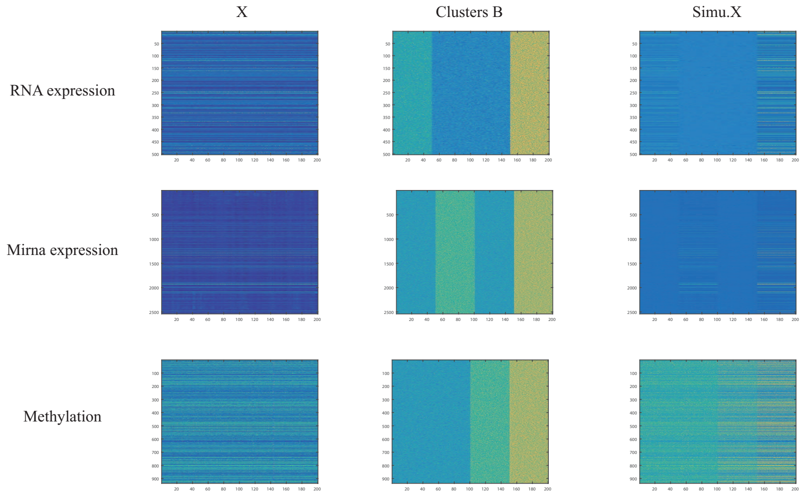

Figure S1.

2.2 Supplementary Figure 2

The surface of NMI for clustered result on SimData1 with respect to hyper-parameter α and β.

2.3 Supplementary Figure 3

The performance of Clusternomics on SimData1 with different noise level (σ from 0.65 to 0.85 at intervals of 0.01), and SimData2 (σ from 0.4 to 0.6 at intervals of 0.01)

2.4 Supplementary Figure 4

The survival curves and the first three principle components of GBM, COAD, and LUSC. It can be noted that clusters derived by HOPES separate clearly no matter in survival duration domain or in spatial distance.

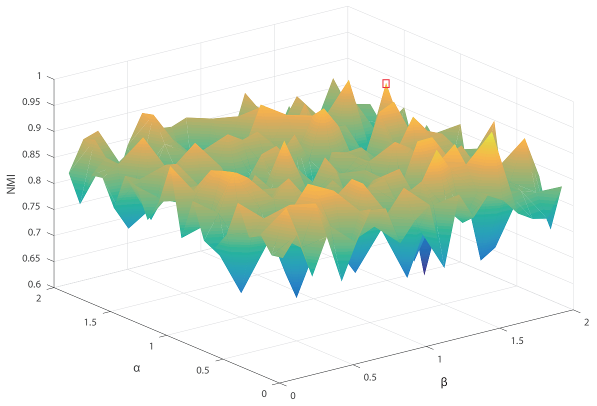

Figure S2.

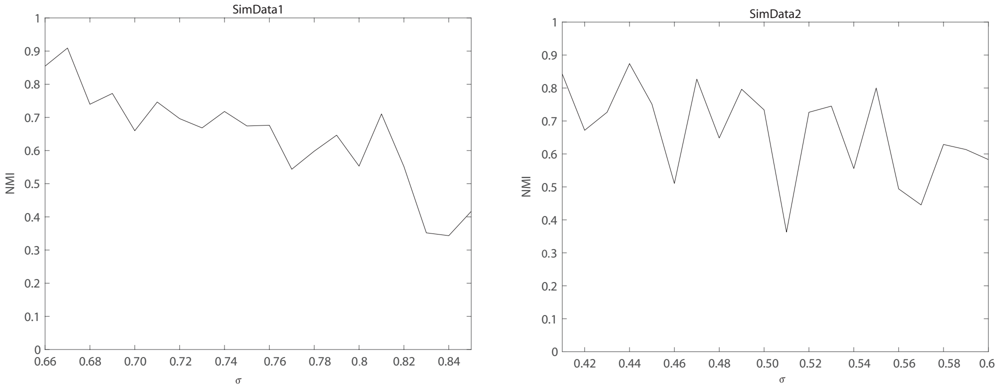

Figure S3.

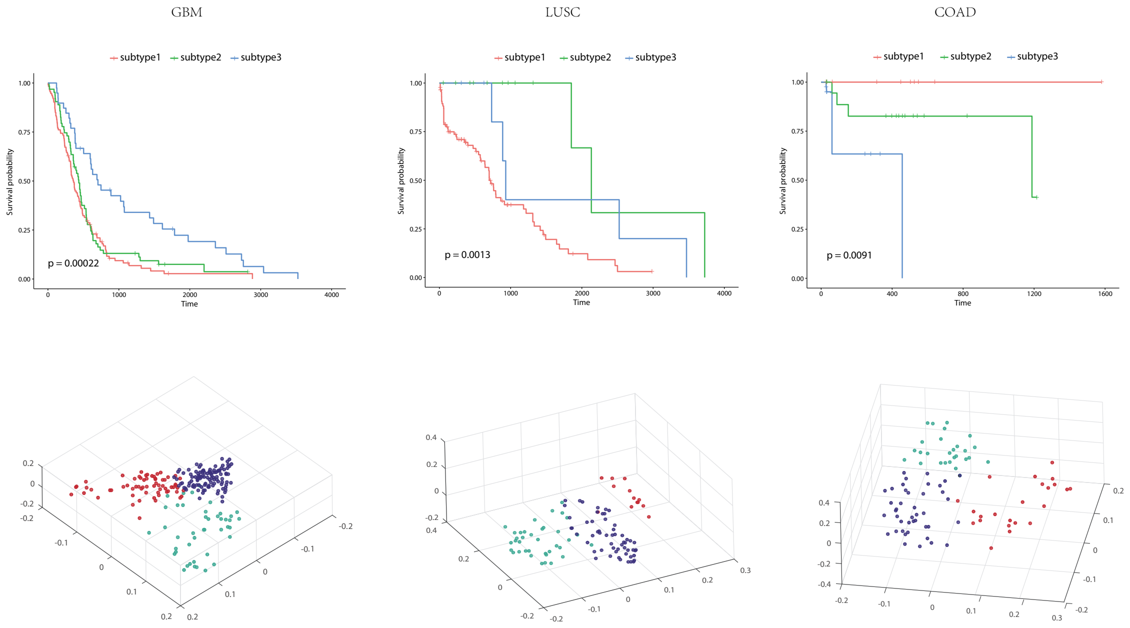

Figure S4.
